# Microbes from ambient-pressure analogues offer insights into possible life in Europa’s high-pressure subsurface ocean

**DOI:** 10.3389/fmicb.2025.1639438

**Published:** 2026-02-12

**Authors:** Alvaro del Moral, Dominic Siggs, Michael C. Macey, Mark G. Fox-Powell, Victoria K. Pearson, Karen Olsson-Francis

**Affiliations:** 1 AstrobiologyOU, School of Environment, Earth and Ecosystem Sciences, Open University, Milton Keynes, United Kingdom; 2 AstrobiologyOU, School of Physical Sciences, Open University, Milton Keynes, United Kingdom

**Keywords:** icy moons, biosignatures, habitability, astrobiology, extremophiles, ocean worlds

## Abstract

Under its thick ice layer, Europa contains a shielded liquid water ocean where habitable conditions may exist. To effectively assess the habitability of this environment and the implications on putative biosignature formation, it is essential to integrate our understanding of the physicochemical conditions of the sub-surface ocean with ground-truth analysis on Earth, using both natural analogue sites and laboratory simulation experiments. This combined approach is particularly prudent for Europa, as locations proposed as natural analogues for the chemistry of Europa’s ocean are predominantly located at ambient pressure (~0.1 MPa), which differs even from the shallowest depths of Europa’s ocean (e.g., 20 to 30 MPa). Basque Lake No. 2, British Columbia, Canada, was used as geochemical analogue for the ice shell-ocean interface and sub-ice environment of Europa due to the Mg-Na-SO_4_ chemistry (maximum 30 to 40% salinity in the summer) and temperature extremes [can reach −45 °C at night in the winter. In this study, microorganisms from the site were grown at elevated pressures in fluid medium based on a model of Europa’s ocean chemistry, mimicking the conditions at Europa’s upper ocean. Following incubation at successively higher pressures, (0.2, 10, 20 and 30 MPa) a microorganism with 99.1% 16S rRNA gene sequence homology to Pseudodesulfovibrio aespoeensis was isolated at 30 MPa (designated Pseudodesulfovibrio sp. OU_01). To our knowledge, this is the first study to demonstrate that microorganisms from an analogue site located at ambient pressure can grow at elevated pressures associated with Europa’s upper ocean.

## 1 Introduction

There is compelling evidence that a sub-surface ocean exists beneath Europa’s ice shell with regions that could fall within the physicochemically-determined habitability limits. Data from Galileo’s magnetometer suggested that the disturbance of Jupiter’s magnetosphere around Europa could not be caused by the moon’s small iron core but by eddy currents generated by the conductivity of a salty liquid water ocean (Khurana et al., 1998; Kivelson et al., 1997, 2000; Kuskov and Kronrod, 2005). Geological features such as the presence of chaos regions (Greenberg et al., 1999) and cycloidal ridges (fractures on the ice shell) (Smith et al., 1979) further corroborate the presence of a liquid water ocean and its interaction with the icy surface. The ocean is likely to be in direct contact with Europa’s silicate mantle, allowing for water-rock reactions to occur, and allowing for water-rock reactions to occur and allowing for creating a potential for chemical disequilibrium (Altair et al., 2018). Radiolysis occurring at the surface could be a potential source of oxidants, which, when mixed with reduced compounds from water-rock reactions, could be used as an energy source by chemolithotrophic microorganisms (Hesse et al., 2022; Russell et al., 2017; Weber et al., 2023).

Measurements from the Galileo magnetometer and radio Doppler data suggest that the salinity of the liquid ocean is similar to that of Earth’s oceans (Khurana et al., 1998; Kivelson et al., 1997, 2000; Anderson, 1998; Trumbo et al., 2019). The temperature of the Europan ocean is predicted to range from 0 °C to 4 °C, with possibly hotspots of higher temperatures (e.g., 40 to 400 °C) near regions of putative ocean floor hydrothermal activity (McCollom, 1999; Merino et al., 2019; Vance and Melwani Daswani, 2020; Zolotov and Shock, 2004). The ice shell is predicted to be between 20 and 30 km thick (Howell, 2021), which would correspond to a pressure at the ice-ocean interface of approximately 20 to 30 MPa (Hand et al., 2006; Styczinski et al., 2023; Vance et al., 2007), with pressures reaching 110 to 360 MPa at the ocean floor, depending on the total depth of the hydrosphere (Marion et al., 2003; Vance et al., 2007; Vance and Goodman, 2009; Vance et al., 2016; Melwani Daswani et al., 2021).

Debate surrounds the chemical composition of Europa’s ocean. Early models based on thermodynamic equilibrium (with an assumed chondritic composition of Europa’s rocky interior) suggested an alkaline magnesium sulfate-dominated ocean (pH 6 to 13), and at the time was thought to explain the detection of hydrated sulfates on the surface (Kargel et al., 2000; Zolotov and Shock, 2001, 2004). More recently, telescope-based infrared observations have suggested that Europa’s more pristine endogenous material may reflect a chloride-dominated ocean composition with potentially high inorganic carbon (Trumbo et al., 2019; Trumbo and Brown, 2023; Villanueva et al., 2023). Consistent with this, Melwani Daswani et al. (2021) investigated the origin of water on Europa by modelling internal metamorphic processes, resulting in an ocean fluid dominated by inorganic carbon, sodium and chloride, as well as calcium, sulfate and magnesium (pH 5 to 8).

In addition to the global subsurface ocean, the presence of salts and other solutes should facilitate stable liquid water within Europa’s ice shell, where pressures may be lower than within the underlying ocean. An ice shell that has formed from a salty ocean, as in the case of Europa, will have incorporated a non-zero quantity of salts (Wolfenbarger et al., 2022), which could allow for a fraction of brine liquid to occur even below the freezing temperature.

To effectively assess the biological potential of Europa’s ocean, it is essential to integrate our understanding of the moon’s physicochemical conditions with ground-based analyses on Earth. One approach is to use natural analogue sites with similar physicochemical conditions; for example, sub-ice environments (Achberger et al., 2016; Biele et al., 2002; Davis et al., 2023; Lowell, 2005; Pearce et al., 2013), deep hypersaline anoxic basins (Hallsworth et al., 2007), subglacial lakes (Pearce, 2017) or hydrothermal vent systems, such as Lost City (Weber et al., 2023). Microbes from these environments have been used as analogues for putative life in the sub-surface oceans of the icy worlds (e.g., Dos Santos et al., 2024; Steinle et al., 2018; Wagner et al., 2022). Another approach is to prioritise analogue localities with similar chemical compositions to that expected for Europa. Buffo et al. (2022) identified the Mg-Na-SO_4_ dominant brines of the Basque Lakes, which lie within the Thomspon Plateau in British Columbia, Canada, as a plausible geochemical analogue for the ice shell-ocean interface and sub-ice environment of Europa. Seasonal snowmelt and groundwater flows leach ions from the surrounding rocks, accumulating in the perennial and ephemeral lakes. The region’s hot and arid summer climate causes significant evaporation, increasing the salinity (can reach 30 to 40% salt by weight) (Buffo et al., 2022). During winter, temperatures decrease from −9 °C to −12 °C, and nighttime lows can reach −45 °C, causing extensive ice coverings to form (Buffo et al., 2022; Renaut and Long, 1989). Microorganisms exist within these environments and molecular analysis has shown that the salts are dominated by *Pseudomonadota* (60%), *Bacilliota* (11%), and unclassified bacteria (27%), whilst cultivation studies have isolated strains of *Marinococcus* and *Halobacillus* (Crisler et al., 2019; Fox-Powell and Cockell, 2018; Srivastava et al., 2021).

The similarity in lake chemistry with some models of Europa’s ocean, and the presence of ice cover, make the Basque Lakes a compelling location for understanding habitability and biosignature formation under conditions expected at Europa. However, the site’s ambient pressure (~0.1 MPa) differs markedly from the predicted pressures of even the shallowest depths of Europa’s ocean [e.g., 20 to 30 MPa near the ice-ocean interface (Vance et al., 2007)]. To extrapolate findings from this environment to Europa, laboratory simulations are required to reach the pressures at which liquid water exists in Europa (Curtis-Harper et al., 2018). Similarly, previous work simulating the conditions of Enceladus has demonstrated that the methanogenic archaeon, *Methanothermococcus okinawensis*, can produce CH_4_ when grown in an optimal growth medium, at 5 MPa (Taubner et al., 2018). For Europa, higher pressures are needed, and thus laboratory-based simulation experiments are technically more challenging and require specialised high-pressure chambers (Foustoukos and Pérez-Rodríguez, 2015; Olsson-Francis et al., 2020; Taubner et al., 2018).

The effect of pressures on microorganisms is generally understudied compared to other extreme conditions due to sampling of high-pressure environments on Earth being technically challenging and the requirement for specialised culturing facilities (Oger and Jebbar, 2010). Most microbes that are known to survive and grow at elevated pressure [above 10 MPa (Jebbar, 2015)] are from deep-sea sediments, marine animals, and hydrothermal vents, and a few strains isolated from the shallower depths of the sea. However, no gene/pathway has been identified as unique to high-pressure adaptation, and recent findings suggest that piezophiles (microbes that thrive in high-pressure environments) employ a common adaptation strategy to cope with multiple types of stresses, such as starvation, acid, salt, temperature, oxidation, and osmosis, which may support survival at high pressure (Allemann et al., 2024; Wang et al., 2021). For example, piezophiles accumulate low-molecular-weight osmolytes, mainly organic solutes, and increase the proportion of unsaturated fatty acids in the lipid membranes (Casadei et al., 2002; Oger and Jebbar, 2010; Wang et al., 2021). Recent studies have demonstrated that bacterial strains *Sporosarcina psychrophila* DSM 6497 and *Lysinibacillus sphaericus* LMG 22257, isolated at 0.1 MPa, were able to grow optimally at 7 and 20 MPa, respectively (Wang et al., 2021). Hence, it is plausible that the microorganisms from the Basque Lakes would be able to survive at pressures associated with the ice-ocean interface of Europa.

In the present study, controlled laboratory simulation experiments were performed to examine the hypothesis that microorganisms from a proposed chemical analogue for Europa’s ocean were able to survive and proliferate under pressures associated with the shallow sub-ice region of Europa’s ocean. For this study, microorganisms from Basque Lake No. 2 were grown in a bespoke analogue Europan fluid medium, which was rich in calcium, sodium, sulfate, carbon dioxide and bicarbonate. Experiments were conducted under pressures analogous to the upper region of the sub-surface ocean of Europa [20 MPa to 30 MPa under an ice shell 20 to 30 km thick (Vance et al., 2007)]. The effect of chemistry and pressure on the growth and diversity of the microbial community was monitored, and a member of the dominant genus was isolated. To our knowledge, this is the first study that has demonstrated that microorganisms from a geochemical analogue site can grow under pressures associated with the shallow sub-ice region of Europa’s ocean.

## 2 Materials and methods

### 2.1 Experimental design

A microbial enrichment was established using sediment collected from Basque Lake No. 2, British Columbia, Canada (49°4′40′′ N, 119°34′3′′ W). The sediment sample was collected aseptically from the margin of the lake, using alcohol-disinfected spatulas, during a sampling campaign in September 2018. The sample was collected from the fine sediment (top 10–20 cm), stored in sterile 50 mL Corning^®^ Falcon^®^ tubes (Sigma-Aldrich, Gillingham, United Kingdom), and shipped in cooling boxes at 4 °C to the Open University (UK) within 7 days of sampling. On arrival in the laboratory, the samples were stored at 4 °C until further use.

**Figure 1:**
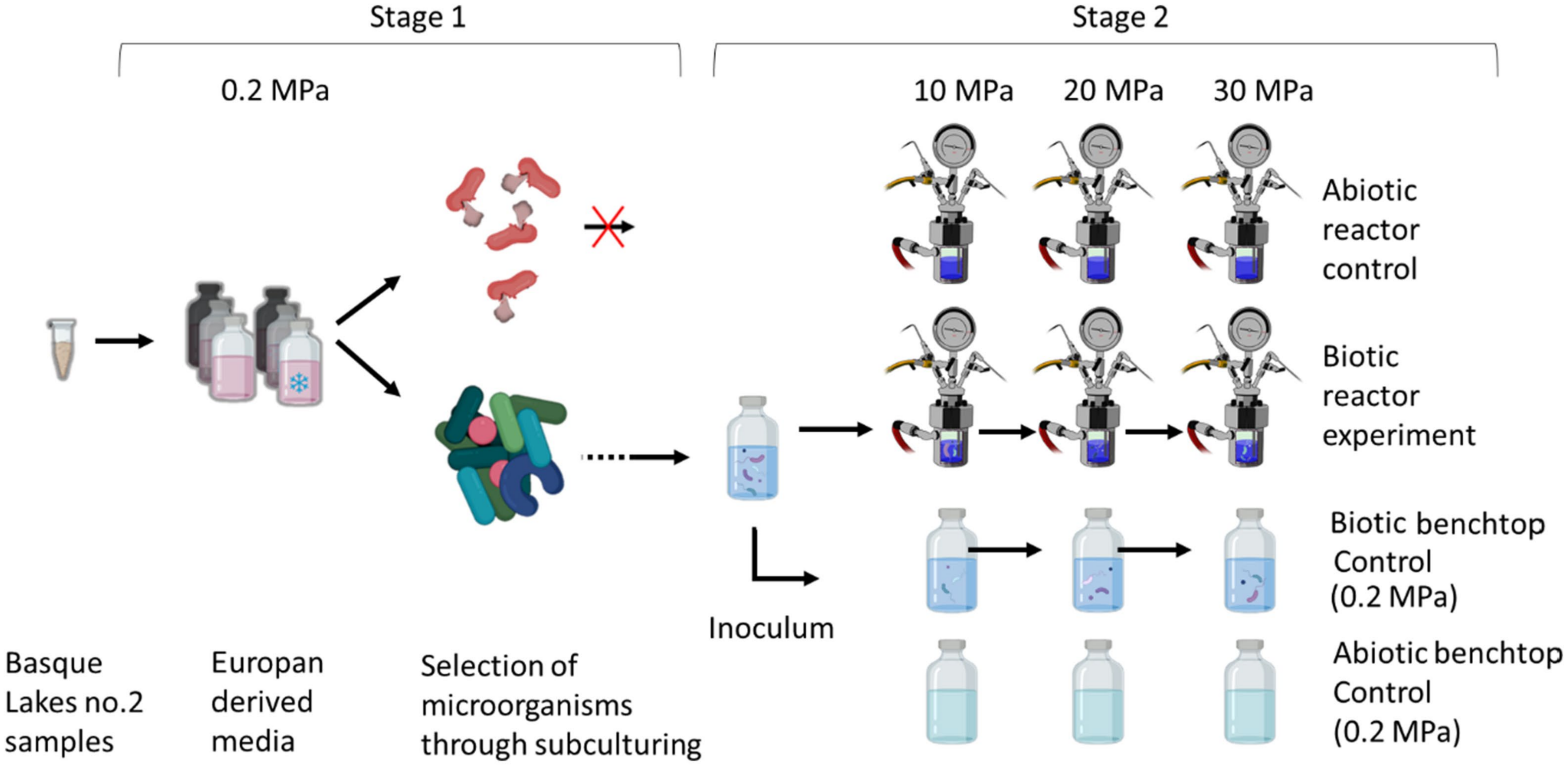
An overview of the experimental design. Stage 1 corresponds to the preparation of the microbial inoculum through eight sequential culturing steps. Stage 2 corresponds to the simulation experiment where the pressure was gradually increased from the initial 0.2 MPa (ambient pressure) to 30 MPa (cells were grown for 12 days at each pressure).

For the enrichment, a slurry was prepared in a 100 mL Wheaton bottle by mixing a 1:1 ratio of sediment and water (25 mL) with the medium under anaerobic conditions. The enrichment was sub-cultured (eight times to remove carryover from the environmental sample) in a simulated Europan fluid medium before inoculating the simulation experiment, as shown in Figure 1. Simulation experiments were conducted at 10, 20 and 30 MPa in a high-pressure reactor, with parallel experiments conducted at ambient pressure. Pressures of 20 and 30 MPa were chosen to simulate conditions in the upper ocean of Europa; 10 MPa was included to allow the microorganisms to adapt through a stepwise increase in pressure. The experiments were conducted at 21 °C (room temperature) and monitored using a thermocouple probe (Platinum RTD Sensor-PT100). Each pressure increase was carried out after briefly (<1 h) depressurising the reactor with a manual valve, to replenish the media. Samples were collected after each step for cell counts, 16S rRNA gene sequencing, and transmission electron microscopy (TEM).

### 2.2 Simulated European fluid medium

An analogue Europan fluid medium was developed based on the ionic species concentration of the model from Melwani Daswani et al. (2021). This model varies with depth, so the composition at 0.2 MPa (which was the lowest pressure used to cultivate the microorganisms) was selected to create the medium. The medium (in detail below) was low in salinity and dominated by NaCl, NaHCO_3_ and SO_4_^2−^ (22.4 mM). Hydrogen (80%) headspace was added as an energy source, for chemolithotrophs as it may be produced by the dissociation of water in the water ice (Szalay et al., 2024) or by geothermal activity at the ocean floor, for example, through serpentinization (Lang et al., 2010; Martin and Russell, 2007). Acetate was added as an additional carbon source to enable chemoorganotrophic metabolism; this may be present in the Europa ocean due to geothermal activity at the ocean floor (Lang et al., 2010; Martin and Russell, 2007).

The medium was supplemented with nitrogen, phosphate, and organic carbon, and contained per L^−1^: 4.23 g of NaCl (Sigma-Aldrich^®^); 4.21 g of NaHCO₃ (Honeywell); 1.43 g of CaSO_4_·2H_2_O (ACROS organics); 1.28 g of MgSO_4_·7H_2_O (ACROS organics); 0.96 g of Na_2_SO_4_ (Fisher Scientific^TM^); 0.51 g of KCl (Fisher Scientific^TM^); 3.45 g of Na_2_SiO_3_ (Sigma-Aldrich^®^); 1.03 g of Na_2_CO_3_ (Fisher Scientific^TM^); 0.28 g of NaHS (Sigma-Aldrich^®^); 42.78 mg of NH_4_Cl (Sigma-Aldrich^®^); 7.10 mg of Na_2_HPO_4_ (Sigma-Aldrich^®^); 8.20 mg of NaCH_3_COO (Sigma-Aldrich^®^) and 11.21 mg of NaC_3_H_5_O (ACROS Organics). Table 1 shows the composition of the Europan fluid medium compared to the modelled values. Preparation was conducted under anaerobic conditions with continuous nitrogen flushing (Price et al., 2022). Filtered sterilised (0.22 μm filter) NH_4_Cl, NaHS, NaCH_3_COO, NaC_3_H_5_O, and 1 mL^−1^ of trace element solution SL 10 (Schlegel et al., 1961), were added to the medium after autoclaving at 121 °C for 10 min.

**Table 1:** The predicted ionic concentrations were derived from the model by Melwani Daswani et al. (2021), along with the final medium composition used to grow microorganisms.

Element	Modelled ocean composition (M)	Europan growth medium (M)
Ca^2+^	1.05 × 10^‑2^	1.05 × 10^‑2^
Cl^‑^	7.95 × 10^‑2^	7.92 × 10^‑2^
CO_3_^‑^	9.74 × 10^‑6^	1.00 × 10^‑5^
Fe^2+^	4.59 × 10^‑15^	0
H_4_SiO_4_	2.83 × 10^‑5^	0
HCO_3_^‑^	5.01 × 10^‑2^	5.01 × 10^‑2^
HS^‑^	4.96 × 10^‑6^	0
K^+^	6.89 × 10^‑3^	6.89 × 10^‑3^
Mg^2+^	5.18 × 10^‑3^	5.18 × 10^‑3^
Na^+^	1.36 × 10^‑1^	1.36 × 10^‑1^
SO_4_^2‑^	2.24 × 10^‑2^	2.24 × 10^‑2^
pH	6.22	6.22
NH_4_Cl	—	8.0 × 10^‑4^
NaCH_3_COO	—	1.0 × 10^‑4^
NaC_3_H_5_O_3_	—	1.0 × 10^‑4^
Na_2_HPO_4_	—	5.0 × 10^‑5^

The modelled value for Fe^2+^ was omitted from the media recipe due to its low concentration (4.6 × 10^−15^ M). Iron was supplied in the SL-10 solution without modification. H_4_SiO_4_ was omitted due to its low solubility and microbial relevance.

For the enrichment, a slurry was prepared in a 25 mL Wheaton bottle by mixing a 1:1 ratio of sediment with the medium under anaerobic conditions. After inverting the Wheaton bottles several times to produce a homogeneous mixture, the simulated Europan fluid medium was inoculated with 5% (vol/vol) of the slurry (*n* = 3). Once inoculated, the Wheaton bottles were sealed, and the headspace was over-pressurised to 0.2 MPa with sterilised gas (80% H_2_ and 20% CO_2_) (Compressed Gas Solutions Ltd.). After 28 days of incubation at 15 °C, the enrichment was sub-cultured to remove carry-over contaminants from the initial slurry and 3 mL were stored at −80 °C for DNA analysis. After sub-culturing the enrichment eight times every 28 days, the culture was used as the initial inoculum for the Europa simulation experiments.

### 2.3 Europa simulation experiments

Simulation experiments were conducted at 10, 20 and 30 MPa (*n* = 1) to cover a range of pressures relevant to the upper ocean of Europa and its ice-ocean interface. Each pressure step was maintained for 12 days (by 12 days, cells were in exponential growth, see Supplementary Figure 1 for details). Focus was kept on the chemistry and pressures of Europa, running each experiment at room temperature, which allowed for the monitoring of biological activity within the experimental timescales. For this, a high-pressure reactor made from Hastelloy^®^ (Alloy C-276) (Haynes international Ltd.) was used, as described in Olsson-Francis et al. (2020). To clean the reactor, a three-stage cleaning and sterilization process was carried out before use to ensure sterility of the internal chamber. The initial step included an inorganic cleaning step to remove salts and particulates. This involved adding 100 mL of ddH_2_O to the chamber and heating it to 160 °C for 24 h. This was followed by an organic cleaning step using Isopropyl alcohol (90%) to eliminate organic contaminants, e.g., lubricants. For the final sterilization step, the chamber was closed, and the temperature and pressure were raised to 121 °C and 0.103 MPa, respectively, for 20 min. The gas lines were cleaned separately with isopropyl alcohol (90%) and autoclaved (121 °C, 15 min). Before use, the lines were purged with N_2_ at 0.2 MPa to remove any particulates.

For the experiment, 380 mL of the Europan fluid medium was prepared and poured into the chamber under a N_2_ gas positive pressure to prevent oxygen and microbial contamination. The chamber was pressurised through a connected gas inlet line with H_2_/CO_2_ and N_2,_ and a gas intensifier unit was used to increase the pressure to 20 MPa and 30 MPa. Initial experiments were conducted at 10 MPa and inoculated, as shown in Figure 1. The inoculum was transferred into the open reactor using a pipette, under a sterile N_2_ gas flow, and then closed and pressurized. Subsequently, the community grown at 10 MPa was used to inoculate the 20 MPa experiment, and so forth. The reactors were inoculated with a 5% inoculum. In between each pressure step, the pressure was decreased to ambient pressure, and the medium was removed with a pipette. 5% (20 mL) of the culture remained in the reactor as an inoculum and then (380 mL) of fresh medium was added. Depressurization was carried out (30–60 min) by opening a manual valve at intervals, preventing sharp drops in temperature. The reactor remained depressurized for less than an hour. Pressure was increased over a 15 to 60 min period to avoid a sharp change in temperature. For controls, the microbial community was grown in Wheaton bottles at 0.2 MPa at room temperature. The control community was sub-cultured and analysed in Wheaton® Bottles, in parallel to the reactor experiments. Abiotic controls were also carried out in parallel in a separate reactor, to monitor for any contamination during the experiment.

Microbial growth, morphology and community diversity were analysed every 2 days. Samples were collected from the reactors into sterilised 100 mL Duran^®^ bottles with bromobutyl rubber stoppers, pre-flushed with sterilised N_2_ gas for 5 min. A sampling line from the chamber was attached to the bottle using a sterilised needle. Two safety, one-way valves were connected to the rubber stoppers to avoid accidental over-pressurization of the bottles. To sample the chamber, the sampling line valve was opened, allowing the fluid to reach the Duran^®^ bottle. The sampling line length and fluid viscosity caused the pressure to drop before reaching the Duran^®^ bottle. The control Wheaton Bottles were sampled anaerobically using a syringe and sterile needles. All samples were stored at −80 °C for DNA extraction and fixed with glutaraldehyde (final concentration of 2.5%) for TEM analysis (Eltoum et al., 2001; Ul-Hamid, 2018).

### 2.4 Monitoring microbial growth

Microbial growth was monitored by direct microscopic counts using a Leica DM2000 LED microscope equipped with a Leica DFC7000 T CCD camera, following staining with SYBR^TM^ Green nucleic acid stain at 1,000× magnification (Invitrogen^TM^), following the modified protocol of Summers et al. (2003). The pre-stained cells were concentrated and immobilized on a 0.45 μM, 47 mm diameter polycarbonate filter using a vacuum-filter system. The filters were treated with anti-fade solution Citifluor AF1 (Sigma-Aldrich^®^) and viewed using a Leica fluorescent microscope at 40× magnification. Enumeration was conducted using ImageJ (Schindelin et al., 2012; Schneider et al., 2012).

### 2.5 Microbial diversity

DNA was extracted from 1 mL of culture using the MP Biomedicals, Inc. FastDNA^™^ Spin kit for soil, per the manufacturer’s instructions. The DNA was quantified using NanoDrop One^™^ and Qubit^™^ (Thermo Fisher Scientific). For sequencing, the V4–V5 region of the 16S rRNA gene was amplified from the extracted DNA using com1 (CAGCAGCCGCGGTAATAC) and com2 (CCGTCAATTCCTTTGAGTTT) primers (Lane et al., 1985), as previously described (Macey et al., 2023). The PCR product was sequenced using the Illumina Miseq Platform by Molecular Research LP (Texas, United States).

Data analysis was performed in R (version 3.9). The package “dplyr” was used for data manipulation and “ggplot” for visualisation (R Core Team, 2022). The DADA2 methodology for Big Data 1.4 was used to analyse the bacterial 16S rRNA Illumina amplicon data in RStudio x64 RStudio 2022.07.2.576 “Spotted Wakerobin” (RStudio Team, 2022) using the R package “dada2” v.1.8.0 (Callahan et al., 2016). The readings were evaluated for quality before filtering based on the anticipated error rate (*E* ≥ 2%, Edgar and Flyvbjerg, 2015). The function “filterAndTrim” was used to do the filtering step, along with the truncation of reads to a length of 220 bp. All sequences containing nucleotides that were not specified (“N”) were manually removed in RStudio. The parametric error model from the DADA2 package was used to calculate the error rates of either the forward or reverse-filtered reads. Prior to merging the forward and reverse reads, error models were applied during the sample inference step on the filtered and trimmed reads to generate fully denoised sequences. All pair-end sequences were merged, and chimeras and sequences less than 150 bp and/or with ambiguous base calls were removed. The function “removeBimeraDenovo” was used to build a “consensus” method for removing chimeras. The abundance of each ASV (sequences differing at least by a single nucleotide) for each sample was then recorded in a data matrix. The SILVA database was used to establish a taxonomy (release 138.2, Quast et al., 2013). The datasets generated for this study can be found in the NCBI Sequence Read Archive with the PRJNA1270039 BioProject number.

Based on the data acquired from the “blank” samples, the ASV table was further modified by a sample decontamination step that was carried out with the package “decontam” (Davis et al., 2018) and 0.0088% of sequences were identified as potential contaminations and eliminated (i.e., DNA extraction blanks and experimental controls). Any ASV identified as belonging to Archaea or Eukaryota (including mitochondria and chloroplasts, 7.2%) were eliminated during this stage. The package “phyloseq” was used to process the sequences (McMurdie and Holmes, 2013). The results were then grouped according to the experimental pressures of each run, and the taxonomic profiles illustrated graphically *via* a barplot, showing relative abundances of each genus level. Those taxa present at relative abundances of <1% were collected in the group “Other”. Sequences for which there were no matches in the database were labelled “Unidentified.”

### 2.6 Cell morphology

Cell morphology was monitored using a Leica fluorescent microscope at 40× magnification, as described above, and with a transmission electron microscope (TEM) [JEM 1400 TEM (Jeol)]. The cells were fixed with 5% glutaraldehyde (1:1 ratio) and stored at 4 °C (Eltoum et al., 2001; Ul-Hamid, 2018). The resulting resin blocks were sectioned (100 nm thick) with a diamond saw and collected onto a copper grid coated with a pioloform support film, which was stained to enhance contrast in 3% uranyl acetate (30 min) and Reynolds lead citrate (Reynolds, 1963). The sections were imaged at an acceleration voltage of 80 kV (Olsson-Francis et al., 2010).

### 2.7 Microbial isolation

For isolation, the microbial community, which had grown at 30 MPa, was grown in a modified European fluid medium at 0.2 MPa to select for the dominant microbial group (sulfate-reducing bacteria). The medium contained per L^−1^: NaC_3_H_5_O_3_ 6.39 g; NaCl 4.23 g; CaSO_4_·2H_2_O 1.43 g; MgSO_4_.7H_2_O 1.28 g; NH_4_Cl 1 g; yeast extract 1 g; Na_2_SO_4_ 0.96 g; KCl 0.51 g; FeSO_4_·7H_2_O 0.50 g; KH_2_PO_4_ 0.50 g; NaCH_3_COO 0.33 g; Na_2_CO_3_ 0.10 mg; NaHCO_3_ 4.21 g. The reducing agent was substituted with sodium thioglycolate (NaHSCH_2_COO 0.1 g L^−1^) and ascorbic acid (C_6_H_8_O_6_ 0.1 g L^−1^). The pH was adjusted using 1 M NaOH to 7.5. A 10% inoculum was used and the cultures were over-pressurised to 0.2 MPa with sterilised gas (80% H_2_ and 20% CO_2_). The cultures were incubated for 12 days at 21 °C and growth was monitored by the production of H_2_S that turned the media from clear to black. Pure cultures were obtained from serial dilution and confirmed using a Leica fluorescent microscope at 40× magnification, as described above.

### 2.8 Molecular identification

The isolate was identified *via* genome sequencing. DNA was extracted from the pure culture using the FastDNA^™^ Spin kit for soil (MP Biomedicals, Inc.) per the manufacturer’s instructions. Whole genome sequencing was carried out by MicrobesNG. Genomic DNA libraries were prepared using the Nextera XT Library Prep Kit (Illumina, San Diego, United States) and sequenced on an lllumina NovaSeq 6000 (Illumina, San Diego, United States) using a 250 bp paired end (Bankevich et al., 2012; Bolger et al., 2014). Coverage of 53-fold was achieved, calculated using BWA, SAMtools (v0.1.19), and BEDTools genomecov (v2.2.7) with default settings (Li et al., 2009; Li and Durbin, 2009; Quinlan and Hall, 2010).

Raw reads were trimmed using Trimmomatic (v0.30; Default settings) (Bolger et al., 2014), and *de novo* assembly was carried out with SPAdes (v3.7; default settings) (Bankevich et al., 2012). Genome completeness and contamination was assessed with CheckM (v1.4.0; Default settings) (Parks, 2015). Taxonomy of the assembled genome was confirmed *via* Protologger and the Type Strain Genomic Server (Meier-Kolthoff and Göker, 2019; Hitch, 2021). Genome annotation was performed using the Rapid Annotations Subsystems Technology (RAST) annotation server (v2.0) with the classic RAST pipeline (Aziz, 2008). A neighbour-joining tree was calculated using complete 16S rRNA gene sequences from the isolate and other *Desulfovibrionaceae*. The phylogenetic tree was built using MEGA version 12.0. The evolutionary distances were calculated using the Neighbour-Joining method default options, and the bootstrap method for 1,000 replicates. The Whole Genome Sequencing (WGS) can be found in the NCBI database under BioProject PRJNA1270039 and submission project SUB15726660.

## 3 Results

### 3.1 Microbial growth

There was no significant difference in the abundance of microbial cells following incubation in the initial sub-culture step (Stage 1) (*p*-value 0.34). After 12 days, cell numbers varied between 9.2 × 10^6^ cells mL^−1^ (1st sub-culture) to 2.2 × 10^7^ cells mL^−1^ (8th sub-culture). The 8th sub-culture was used to inoculate the 10 MPa simulation experiment, and subsequently, after 12 days, the 10 MPa culture was used to inoculate the 20 MPa experiment and so on. When sampled at the beginning of each pressure step, the initial cell concentration at 10 MPa was 2.08 × 10^5^ ± 5.66 × 10^4^ cells mL^−1^, at 20 MPa was 3.46 × 10^5^ ± 3.63 × 10^4^ cells mL^−1^, and at 30 MPa, was 3.70 × 10^5^ ± 1.36 × 10^4^ cells mL^−1^. The biotic controls at 0.2 MPa were inoculated in parallel, as shown in Figure 1, from an initial concentration of approximately 10^5^ cells mL^−1^.

After 12 days of incubation, the cell concentrations were 8.11 × 10^7^ ± 2.2 × 10^7^ cells mL^−1^ at 10 MPa, 4.59 × 10^7^ ± 1.84 × 10^7^ cells mL^−1^ at 20 MPa, and 1.24 × 10^7^ ± 0.44 × 10^7^ cells mL^−1^ at 30 MPa and were reached during stationary phase. In comparison, the maximum cell concentrations in the biological controls were 5.80 × 10^7^ ± 2.08 × 10^7^ cells mL^−1^, 8.76 × 10^7^ ± 2.76 × 10^7^ cells mL^−1^, and 1.07 × 10^8^ ± 4.72 10^7^ cells mL^−1^. There were no significant differences (ANOVA test using the function “aov” in base RStudio) between the total cell counts of each high-pressure experiment. No cells were observed in the abiotic controls.

### 3.2 Microbial diversity

**Figure 2:**
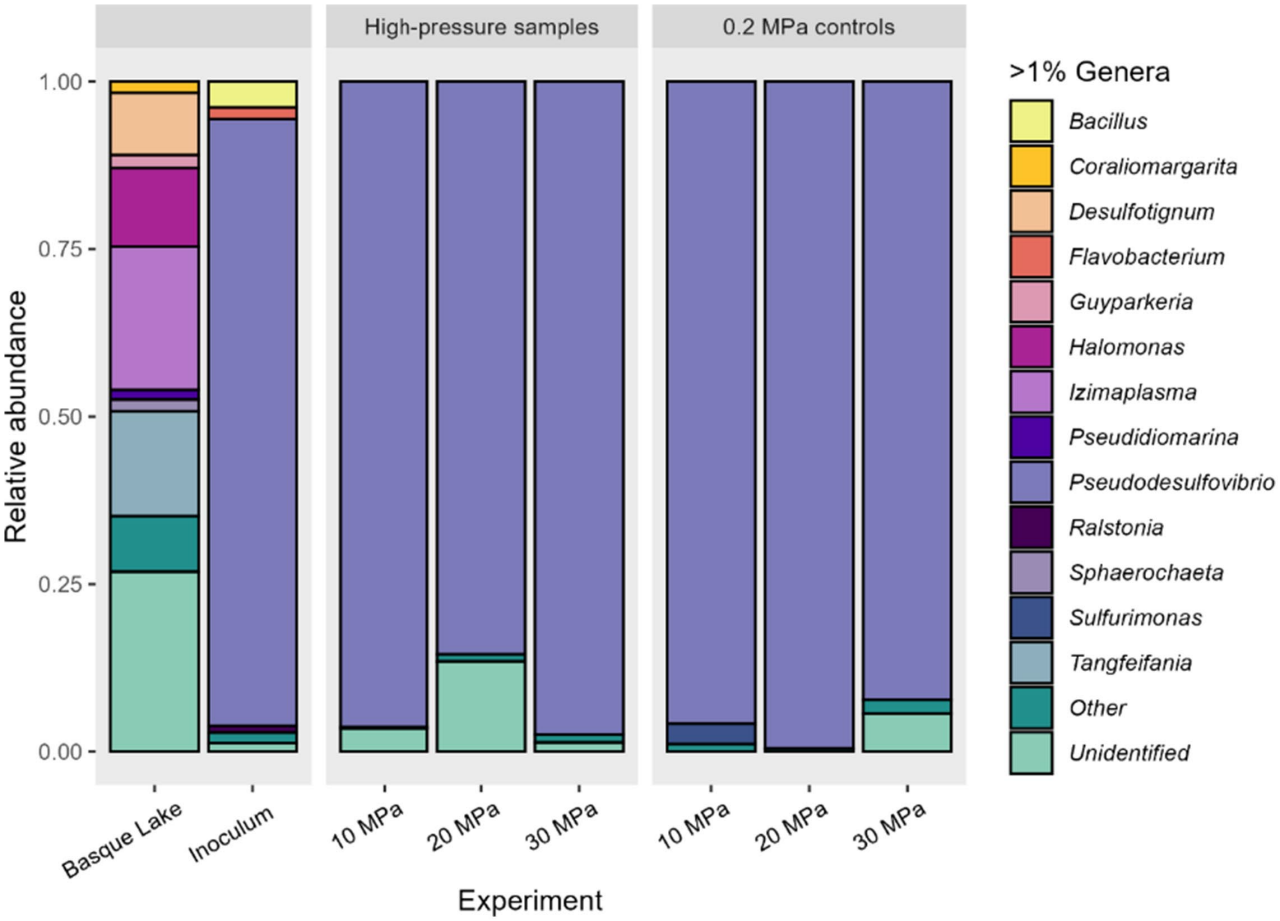
Barplot of relative abundances of identified taxa at the genus level using 16S rRNA gene sequencing data. “Other” represents minor taxa below 1%. Results for the experimental data from the high-pressure reactors, the lower pressure controls (0.2 MPa), the initial sample from Basque Lake, and the final subcultured community in Europan fluid media used as an inoculum for the high-pressure experiments.

Microbial diversity was monitored at each pressure by sequencing 16S rRNA gene amplicons. A total of 122,483 raw sequences from the sediment, environmental simulations and controls were analysed, resulting in a total of 102,027 reads (after quality control). The rarefaction curve reached a plateau, indicating the sequencing depth was adequate (Supplementary Figure 2). As demonstrated in Figure 2, the most relevant abundant genera in the initial sediment were *Izimaplasma* (21.34%), *Tangfeifania* (15.65%), *Halomonas* (11.73%), *Desulfotignum* (9.28%), *Guyparkeria* (1.96%) and *Sphaerochaeta* (1.44%). Alpha-diversity analyses (Supplementary Figure 3) demonstrated a substantial difference in diversity between the sediment and inoculum used in the simulation experiments (sub-cultured eight times in the Europa fluid medium). For example, the Shannon index for the sediment was higher (Shannon index of 3.59) compared to the inoculum, which was 0.849. The *Pseudoesulfovibrio* genus, which represented 0.62% relative abundance of the initial community, dominated the inoculum community (90.23%) and remained above 85% in the simulation experiments (10, 20 and 30 MPa) and the benchtop controls. The lack of diversity was reflected in the Shannon index, and it remained constant at 1.07 ± 0.23 in the simulation experiments (Supplementary Figure 3).

### 3.3 Microbial morphology

**Figure 3:**
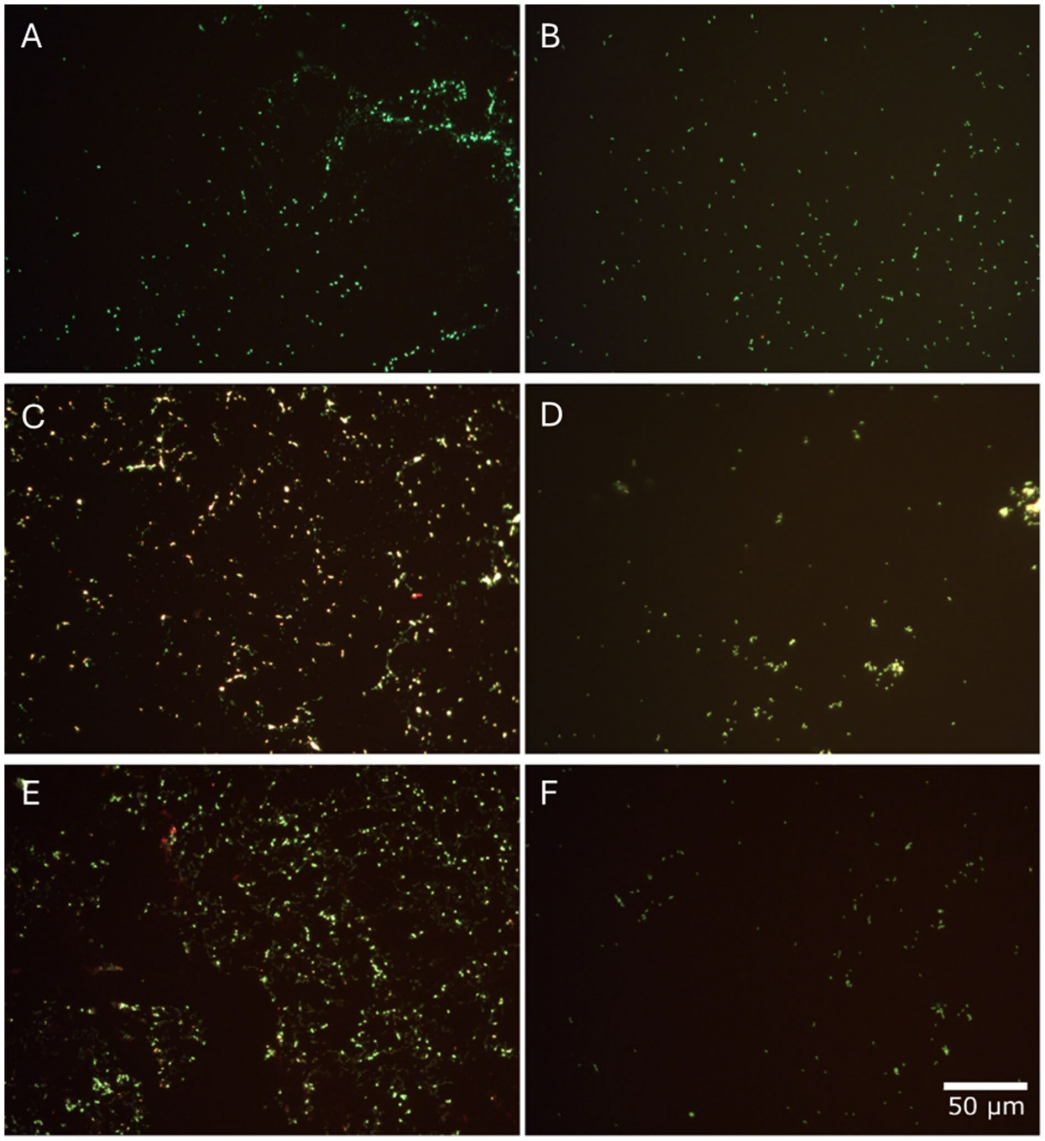
Micrographs of acridine-orange-stained cells under a 40× magnification (scale bar applies to all). Rows correspond to each pressure experiment: 10 MPa, reactor sample **(A)** and its room pressure control **(B)**; 20 MPa, reactor sample **(C)** and its room pressure control **(D)**; 30 MPa, reactor sample **(E)** and its room pressure control **(F)**.

Fluorescent microscope images show the microbial cells stained with Live/Dead stain after 12 days of growth (Figure 3). For cells grown at 10 MPa (Figure 3A), 20 MPa (Figure 3C) and 30 MPa (Figure 3E), extracellular material or cellular debris, and clumped cells were observed. These structures were not observed in the benchtop biological controls (Figures 3B,D,F). The inoculated control and the biotic control presented no differences. The cell numbers were not representative of the distribution of cells across all fields of view (FOVs), with cells often clumping in some areas, which is shown in Figure 3.

**Figure 4:**
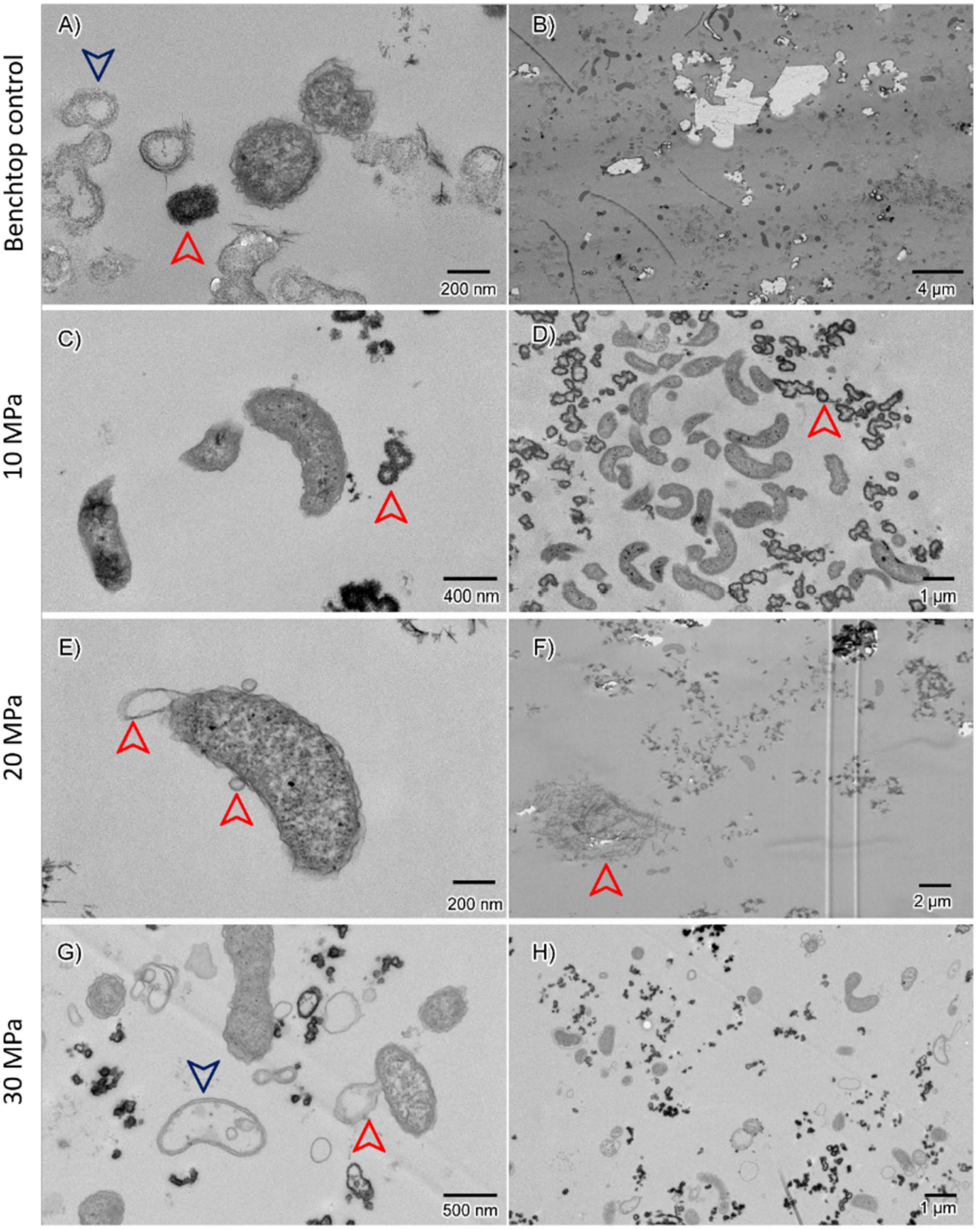
Transmission electron microscope images at low and high magnifications of cells grown under different pressures **(A,B)** belong to the room pressure control experiment (0.2 MPa); **(C,D)** to the 10 MPa experiment; **(E,F)** to the 20 MPa experiment; **(G,H)** to the 30 MPa experiment. Red and blue arrows point structures discussed in the text.

TEM images (Figure 4) demonstrated that the community was dominated by microorganisms with rod-like morphologies, presenting an undulated outer cell wall and accompanied by unidentified extracellular organic material. Cell morphologies were similar across all experiments. Some cells showed signs of damage, including specific structures, such as vesicles or empty cell walls, when compared to healthy cells.

For the benchtop biological controls, several intact cells and the unidentified organic material were seen (Figure 4). However, no apparent damaged cells were observed. In a few instances, smaller and denser objects were found, possibly spores; for example, in Figure 4A, where dark grey cells are seen (red arrow) next to rod-cells cut transversally. The rod-like morphologies are clearly seen in Figures 4C,D (grown at 10 MPa). The curved shape and undulated cell wall match images in the literature of *Desulfovibrio*, which corresponds to the sequencing data.

In Figure 4F, cells grown at 20 MPa were surrounded by organic material (red arrow), this time presenting a different structure, resembling a bundle of needles. The effect of pressure on the cell becomes apparent, for example, in Figure 4E, with the formation of vesicles (red arrows). As the pressure increased to 30 MPa (Figures 4G,H), damaged cells were observed, showing the characteristic formation of vesicles (red arrow) as well as empty cells where only the cell wall remains (blue arrow).

### 3.4 Molecular identification

**Figure 5:**
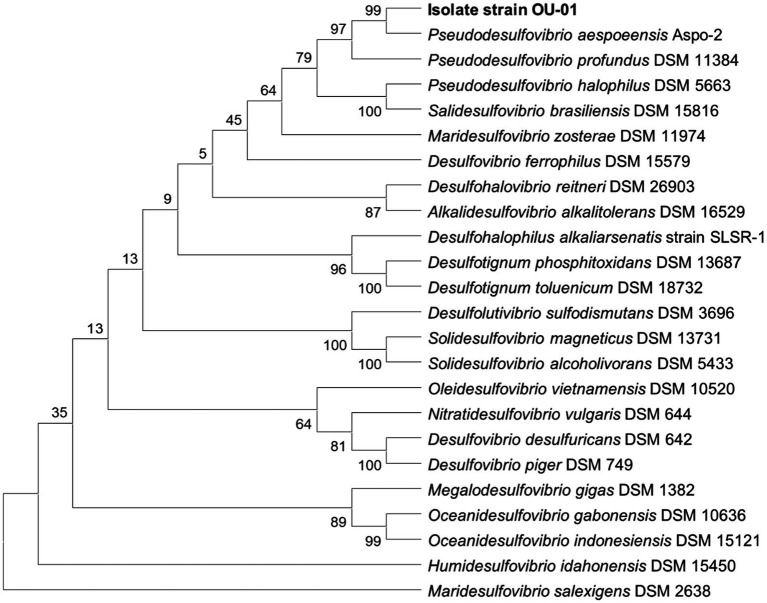
Neighbour-joining tree of 16S rRNA gene sequence similarity from 1,000 bootstrap replicates, showing the consensus phylogenetic position of strain OU_01 and representative of the family *Desulfovibrionaceae*. The sequence of *Maridesulfovibrio salexigens* was used as an outgroup.

The assembled genome sequence yielded 3,283,180 bp distributed in 65 contigs with 62.80% GC content, an N50 of 116,975 bp, a completeness score of 98.82% and a contamination score of 0%. Isolate OU_01 was most closely related to *Pseudodesulfovibrio aespoeensis* (Aspo-2), with 99.1% 16S rRNA gene sequence similarity. Figure 5 shows the phylogenetic positions of strain OU-01, with representatives of the family *Desulfovibrionaceae*. At a genome level, ANI and dDDH scores confirmed the match with *Pseudodesulfovibrio aespoeensis* (Aspo-2) with 71.4% dDDH value with a confidence interval of 67.5–75.1 and 92.46%, respectively.

## 4 Discussion

In this paper, laboratory simulation experiments were performed to examine whether a microbial community from the Mg-Na-SO_4_ dominant brines of the Basque Lake No. 2 (Buffo et al., 2022) could survive and grow at pressures associated with the ice-ocean interface of Europa (10 MPa to 30 MPa). The results show that microorganisms from ambient pressure environments, that are accustomed to high levels of dissolved sodium, magnesium, chloride and sulfate [as predicted for Europa’s ocean (Melwani Daswani et al., 2021)] can grow under pressure conditions representing the upper ocean of Europa.

The results showed that the chemistry of the analogue Europan fluid had a significant impact on the diversity of the microbial community, with members of the genus *Pseudoesulfovibrio* dominating the community (the Shannon index was 0.849 after growth in the Europan fluid medium compared to 3.59 for the Basque Lake No. 2 sediment). Members of the genus continued to dominate the community (above 85% relative abundance, for 2.34 × 10^7^, 1.87 × 10^7^, 1.24 × 10^7^ cells mL^−1^, at 10, 20 and 30 MPa respectively) when grown at pressures associated with the upper region of the sub-surface ocean of Europa (20 MPa to 30 MPa under an ice shell 20 to 30 km thick) (Vance et al., 2007). Due to the microorganisms from the Basque Lakes additionally experience extremes of temperature and salinity in their natural environment, we propose that this high pressure-tolerant isolate is well suited as a model for investigations of microbial habitability and biosignature production under the combined extremes of Europa’s ocean.

Our results indicate that fluid chemistry played a more significant role than pressure in shaping microbial assemblages. However, much of the DNA from unidentified organisms in the environmental sample could belong to unculturable organisms and account for part of the change seen in the community composition. Figure 2 shows that the greatest difference in diversity was observed between the Basque Lake No. 2 sample and the inoculum, in which a community was established in the Europa fluid medium following 8 sub-cultures at ambient pressure. By comparison, the diversity changed little when pressure was successively increased, and the medium chemistry kept constant. Similarly, the 0.2 MPa controls showed little difference in diversity from the high-pressure incubations. The alpha diversity indices (Chao1, Shannon, Simpson and the evenness factor) showed similarity at the different pressures and controls, compared to the original Basque Lake sample (Figure 2). Specific chemical properties of the Europan fluid medium must have promoted the enrichment of the isolate and inhibited the growth of other members of the Basque Lake No. 2 community.

The Phyla Bacillota Bacteroidota, dominated the microbial community in the Basque Lake No. 2 sediment, and are present in other Na- and Mg-sulfate playa brine environments located in China (Han et al., 2017), Russia (Kallistova et al., 2020) and Spain (Cabestrero et al., 2018; Cabestrero and Sanz-Montero, 2018), Spotted Lake (MgSO_4_-rich), Canada (Pontefract et al., 2017) and the United States (Kilmer et al., 2014; Lindemann et al., 2013). By contrast, the Europan fluid medium was Mg-poor relative to Ca^2+^, which may have inhibited some taxa adapted to high Mg^2+^ concentrations, leaving a potential for opportunists with less compositional specificity to grow. Furthermore, the substrates available for metabolic pathways in the Europan fluid media was restricted compared to that likely available in the natural environment, where interaction with the atmosphere and input from local vegetation can provide rich energy sources for heterotrophic and aerobic clades. In the enrichment sub-cultures, the community’s nutritional requirements were met by either compounds dissolved in the medium itself (Table 1) biomass or byproducts of other community members [e.g., exuded organic carbon (Christie-Oleza et al., 2017; Macey et al., 2023; Wright et al., 2019)].

The *Pseudoesulfovibrio* genus dominated the microbial communities at 0.2 MPa and 30 MPa, despite being a minor component of the Basque Lake No. 2 sediment community. The success of this genus in our enrichments demonstrates that *Pseudoesulfovibrio* is capable of acclimatising rapidly to different major ion chemistries.

The functional potential of the isolate provides insight into potential metabolisms that are feasible within Europa-relevant fluids at pressures relevant to the upper ocean of Europa. The genus *Pseudodesulfovibrio* [initially classified as *Desulfovibrio* genus (Cao et al., 2016)] is metabolically diverse and capable of both autotrophic and heterotrophic growth using thiosulfate, sulfate, sulfite, nitrite, nitrate, iron and oxygen as electron acceptors (Krekeler and Cypionka, 1995). Based on the 16S rRNA gene, the dominant isolate from the simulation experiment had a 99.1% homology to *Pseudodesulfovibrio aespoeensis* Aspo-2 (DSM 10631 T), which was isolated from granitic groundwater sampled at a depth of 600 m (6–11 MPa) (Motamedi and Pedersen, 1998). Previous studies have shown that *Pseudodesulfovibrio aespoeensis* can grow lithoheterotrophically using hydrogen and acetate (as the carbon source) with electron acceptors including sulfate, thiosulfate and sulfur. Given the lack of iron, oxygen and nitrate/nitrite in the Europan fluid medium, it is plausible to assume that this was the case in this study. On Europa, a similar metabolic pathway may be plausible, particularly if there is a continuous supply of oxidised sulfur species driven by radiolysis at the surface (Greenberg, 2010; Szalay et al., 2024) with H_2_, acetate and other short-chained hydrocarbons forming as byproducts of serpentinization reactions at the ocean floor (Greenberg, 2010; Hand et al., 2006; Pasek and Greenberg, 2012; Russell et al., 2017).

There is good rationale to consider sulfate reduction a plausible metabolic pathway for life in high-pressure saline environments such as Europa’s ocean. On Earth, microbial sulfate reduction is a ubiquitous process found in numerous anoxic environments, such as deep ocean hydrothermal vents and cold seeps (Kallmeyer and Boetius, 2004; Roerdink et al., 2024). Microorganisms belonging to the genus *Pseudodesulfovibrio*, for example, the reclassified *Pseudodesulfovibrio piezophilus* (Khelaifia et al., 2011), *Pseudodesulfovibrio profundus* (Bale et al., 1997) have been isolated from high-pressure marine environments. Although hydrogenotrophic methanogens (archaea capable of consuming H_2_ to produce methane) are routinely considered as a model microorganism for astrobiology, they are generally outcompeted by sulfate-reducing bacteria (SRB), for molecular hydrogen (Abram and Nedwell, 1978; Smith et al., 2008) and acetate (Oremland and Taylor, 1978) in hypersaline environments.

Although the microorganisms used in this study were isolated from an ambient pressure environment, they were able to survive and grow at 30 MPa. At this pressure, the microorganisms must be able to withstand the adverse impact of hydrostatic pressure on the fluidity and permeability of the cell membrane, DNA replication, cell division, and cellular processes, such as motility and osmotic potential (Abe, 2015; Bartlett, 2002; Eloe et al., 2008; Meganathan and Marquis, 1973; Mota et al., 2013; Picard and Daniel, 2013; Sehrawat et al., 2021). This could be due to common adaptation strategies employed in extreme environments, such as the accumulation of low molecular weight osmolytes, mainly organic solutes, which are responsible for extreme cold, oxidative stress, starvation and salinity responses (Zhang et al., 2015). Many of these solutes offer protective properties, for example, protecting cellular metabolism and scavenging reactive oxygen species generated under stress (Zhang et al., 2015). Previous work has also shown that high pressure and low temperature have the same response on membrane fluidity, causing an increase in the proportion of unsaturated fatty acids in their lipids. We also observed extracellular organic material and vesicle production at elevated pressure. This could be due to the formation of EPS-like material (extra polymeric substances), for example, a *Halanaerobium* strain isolated from shale produced more EPS and clumped when grown above 0.1 MPa (e.g., Booker et al., 2019). However, this could also be due to the rapid decompression causing cellular damage (Cario et al., 2022; Park and Clark, 2002).

The ability of microorganisms to survive and potentially grow within the upper ocean of Europa would require adaptation to extremes of other parameters in addition to high pressures. For example, temperature will be close to the freezing point, and, for microorganisms at the ice-ocean interface, salinities could also be high as dissolved salts are excluded from forming ice (Buffo et al., 2020). It is reasonable to expect that the isolate obtained in this study, which we have shown can proliferate at the relevant pressures, is well suited to this combination of extremes. In their natural environment, the microbial community would survive seasonally extreme temperatures, as well as high salinities (Buffo et al., 2022; Renaut and Long, 1989). The salinity of the lakes can exceed 300 g L^−1^, due to substantial evaporation and lack of rainfall in the summer (Renaut and Long, 1989). During the winter months, the air temperature decreases and can exceed −45 °C (Buffo et al., 2022; Renaut and Long, 1989), causing substantial ice cover to form. This process further concentrates the underlying brine, depressing its freezing point by as much as −9 °C, depending on the composition of the lake. Previous studies have shown that organisms isolated from these lakes exhibit a high degree of plasticity regarding the range of brine compositions they can tolerate (Fox-Powell and Cockell, 2018), which would suit them well to changes in brine composition that could be experienced in the ice-ocean interface. By selecting members of this community that are capable of growth at high pressures, we have produced an isolate that can be used to investigate the synergistic effects of multiple extremes relevant to life in Europa’s ocean.

Based on the ability of *Pseudodesulfovibrio* sp. OU_01 to grow in an Europan fluid medium at pressures associated with the upper ocean of Europa, it is an ideal model organism with which to study biomolecules that could be used as biosignatures. Future work should focus on understanding how the biomolecular composition of *Pseudodesulfovibrio* sp. OU_01 varies under various combined extremes relevant to Europa’s ocean and ice shell, and how these molecules could be preserved and ultimately detected on Europa’s surface. The surface of Europa is a harsh environment for biomolecules but remains the only region accessible to near-future life-seeking missions. Microorganisms from the ice-ocean interface or trapped in the ice could be delivered to the surface where their remnants or metabolic byproducts are measured. Observations of non-ice materials such as NaCl and CO_2_ in geologically young regions of Europa’s surface provide evidence that transfer from ocean to surface takes place (Trumbo et al., 2019; Trumbo and Brown, 2023). If microorganisms inhabit the upper regions of Europa’s ocean, whole cells or their derivatives may be transported alongside ocean salts, which are well-known to incorporate and preserve molecular biosignatures when frozen (Johnson et al., 2020; Moreras-Marti et al., 2024; Nichols et al., 2023). Furthermore, ice grains expelled from Europa’s surface as dust or in plumes (Becker et al., 2024; Roth et al., 2014; Sparks et al., 2017) could circumvent the need to drill through the ice shell to find biosignatures. Such ice grains could be analysed by instrumentation such as the Surface Dust Analyser (SUDA) (Kempf et al., 2025; Pappalardo et al., 2024), and the Mass Spectrometer for Planetary Exploration (MASPEX) (Grasset et al., 2013; Waite et al., 2024) onboard Europa Clipper, both spacecraft currently heading towards Europa. Laboratory simulation experiments have shown that both instrumentations could detect biosignatures (Chela-Flores, 2022; Chela-Flores, 2017; Dannenmann et al., 2023; Klenner et al., 2022; Napoleoni et al., 2023). However, because both the preservation of biomolecules and their detectability with spacecraft instruments depends on their molecular composition, it is vital to investigate biomolecules from organisms with the greatest relevance to the Europan environment, such as *Pseudodesulfovibrio* sp. OU_01.

## 5 Conclusion and future work

Our research findings demonstrate that microorganisms originating from a proposed chemical analogue for Europa, specifically Basque Lake No. 2 which experiences approximately 0.1 MPa of pressure, were capable of growth in a simulated Europa fluid medium that was subjected to pressures typical of the upper ocean of Europa, ranging from 20 to 30 MPa. Notably, the predominance of the genus *Pseudodesulfovibrio* within this microbial community underscores the potential importance of sulfate reduction as a plausible and significant metabolic pathway that could be utilised by microorganisms in the extraterrestrial environment of Europa.

Moving forward, our future research efforts will concentrate on gaining a deeper understanding of the physiological adaptations that enable these microorganisms to survive and thrive under the selective constraints imposed by pressure, salinity, and temperature specific to the ice-ocean interface. This will involve conducting growth studies using the isolated strain *Pseudodesulfovibrio* sp. OU_01, coupled with comprehensive transcriptomic and genomic analyses. These investigations aim to identify long-term trends in microbial acclimation and adaptation, as well as to discover potential biosignatures that could be used as evidence of life.

## Data Availability

The datasets presented in this study can be found in online repositories. The names of the repository/repositories and accession number(s) can be found at: https://www.ncbi.nlm.nih.gov/bioproject, PRJNA1270039.
