# Supplementary material for: Microbes from ambient-pressure analogues offer insights into possible life in Europa’s high-pressure subsurface ocean
Source: Front Microbiol. 2026 Feb 12;16:1639438. doi: 10.3389/fmicb.2025.1639438 (PMC12938689; doi:10.3389/fmicb.2025.1639438)
Supplement: Supplementary file 1 [file Data_Sheet_1.docx]

# Supplementary Figures

Supplementary Table 1. Values for the predicted ionic concentrations in Melwani Daswani et al., 2021 and the final derived medium used to grow microorganisms. The modelled value for Fe^2+^ was omitted from the media recipe due to its low concentration (4.6 × 10-15 M). Iron was supplied in the SL-10 solution without modification. H_4_SiO_4_ was omitted due to its low solubility and microbial relevance.

| **Element** | **Modelled ocean composition (M)** (Melwani Daswani *et al.*, 2021) | **Europan Growth Medium (M)** |
| --- | --- | --- |
| Ca^2+^ | 1.05 x10^‑2^ | 1.05 x10^‑2^ |
| Cl^‑^ | 7.95 x10^‑2^ | 7.92 x10^‑2^ |
| CO_3_^‑^ | 9.74 x10^‑6^ | 1.00 x10^‑5^ |
| Fe^2+^ | 4.59 x10^‑15^ | 0 |
| H_4_SiO_4_ | 2.83 x10^‑5^ | 0 |
| HCO_3_^‑^ | 5.01 x10^‑2^ | 5.01 x10^‑2^ |
| HS^‑^ | 4.96 x10^‑6^ | 0 |
| K^+^ | 6.89 x10^‑3^ | 6.89 x10^‑3^ |
| Mg^2+^ | 5.18 x10^‑3^ | 5.18 x10^‑3^ |
| Na^+^ | 1.36 x10^‑1^ | 1.36 x10^‑1^ |
| SO_4_^2‑^ | 2.24 x10^‑2^ | 2.24 x10^‑2^ |
| **pH** | 6.22 | 6.22 |


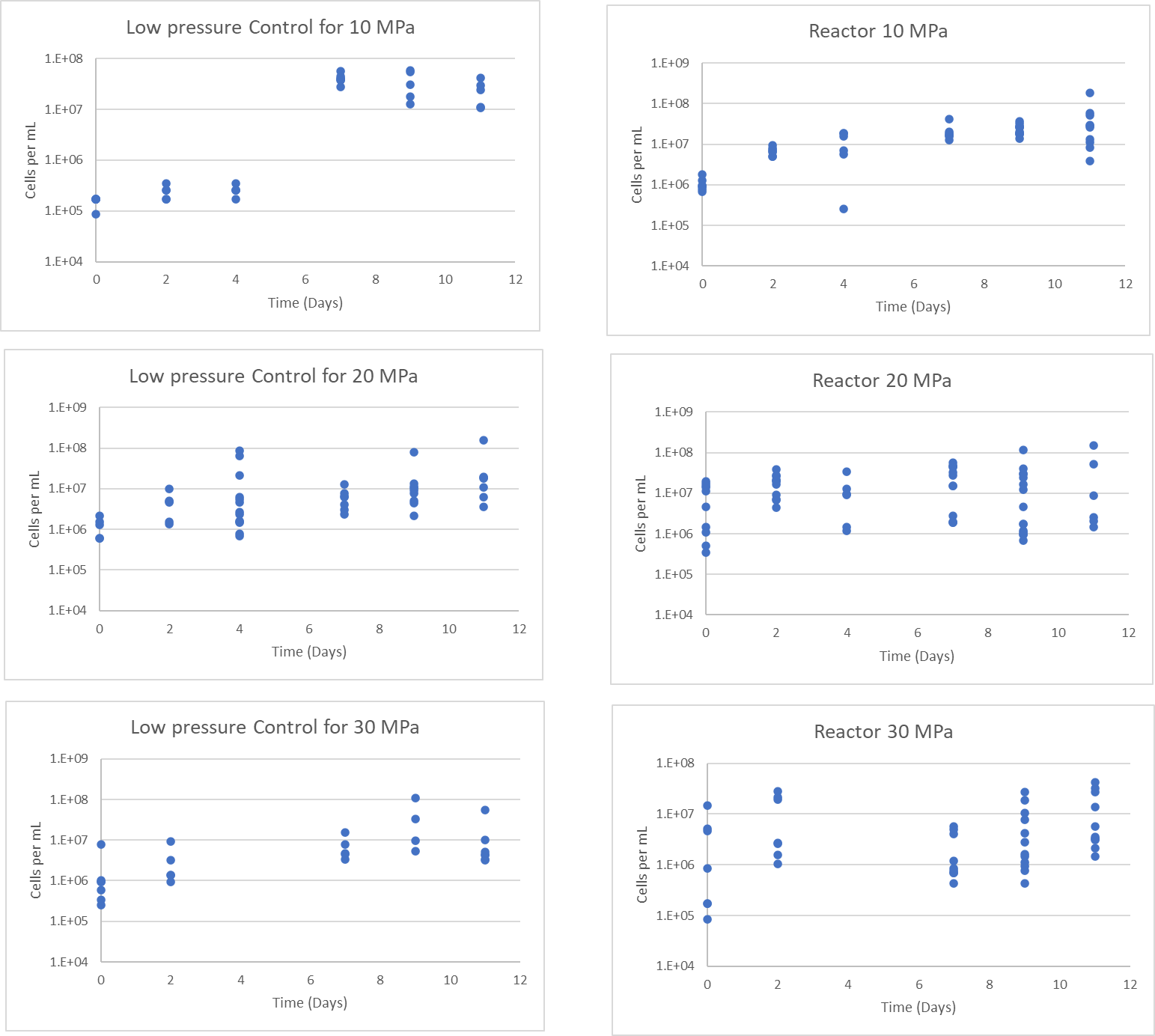


Supplementary Figure 1. Fluorescent microscopy cell counts for the low pressure controls (0.2 MPa) and the corresponding high-pressure reactors.


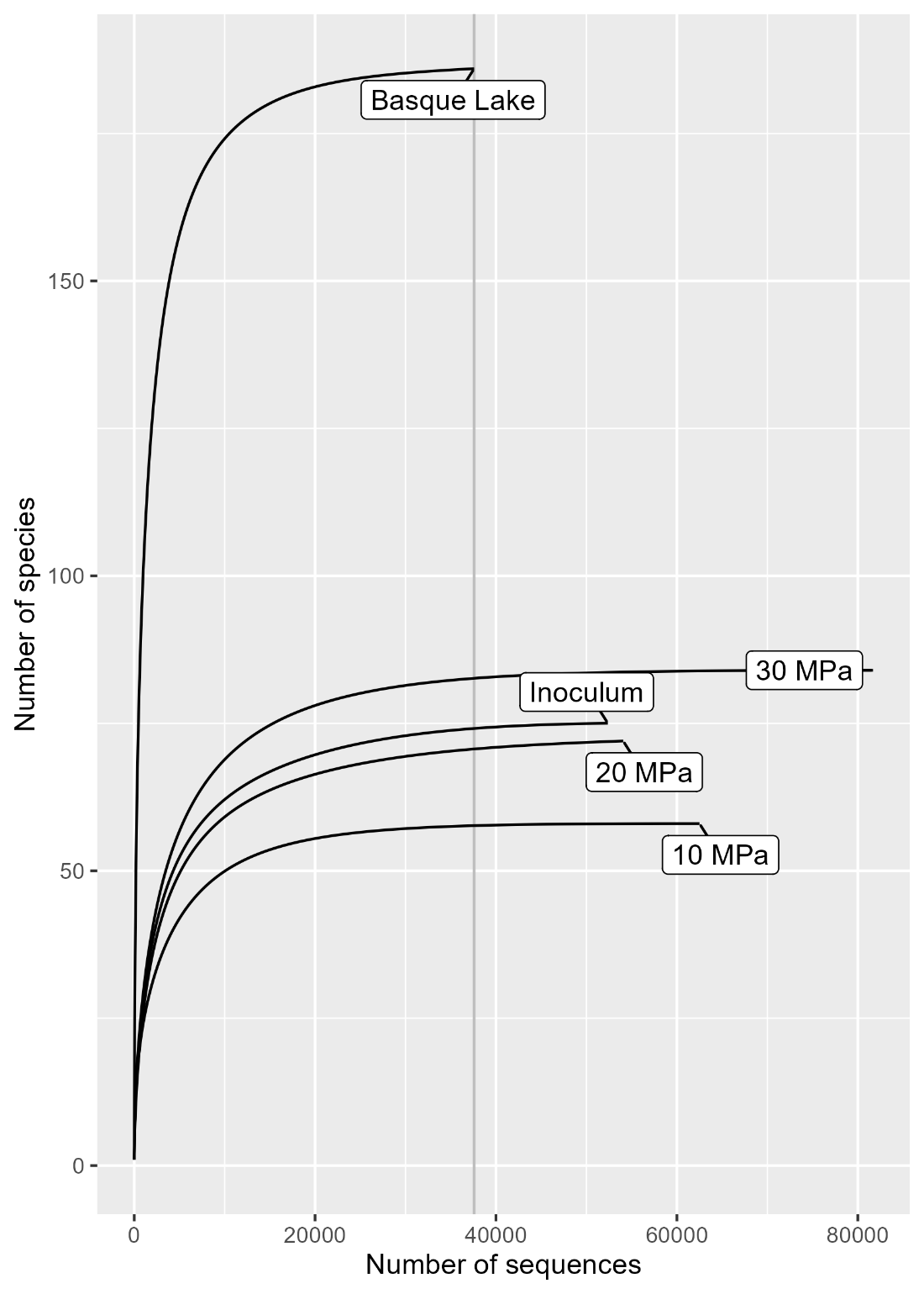


**Supplementary Figure 2 Rarefaction curves showing the diversity as a function of randomized sampling efforts of each of the sequences for each experiment.**

**
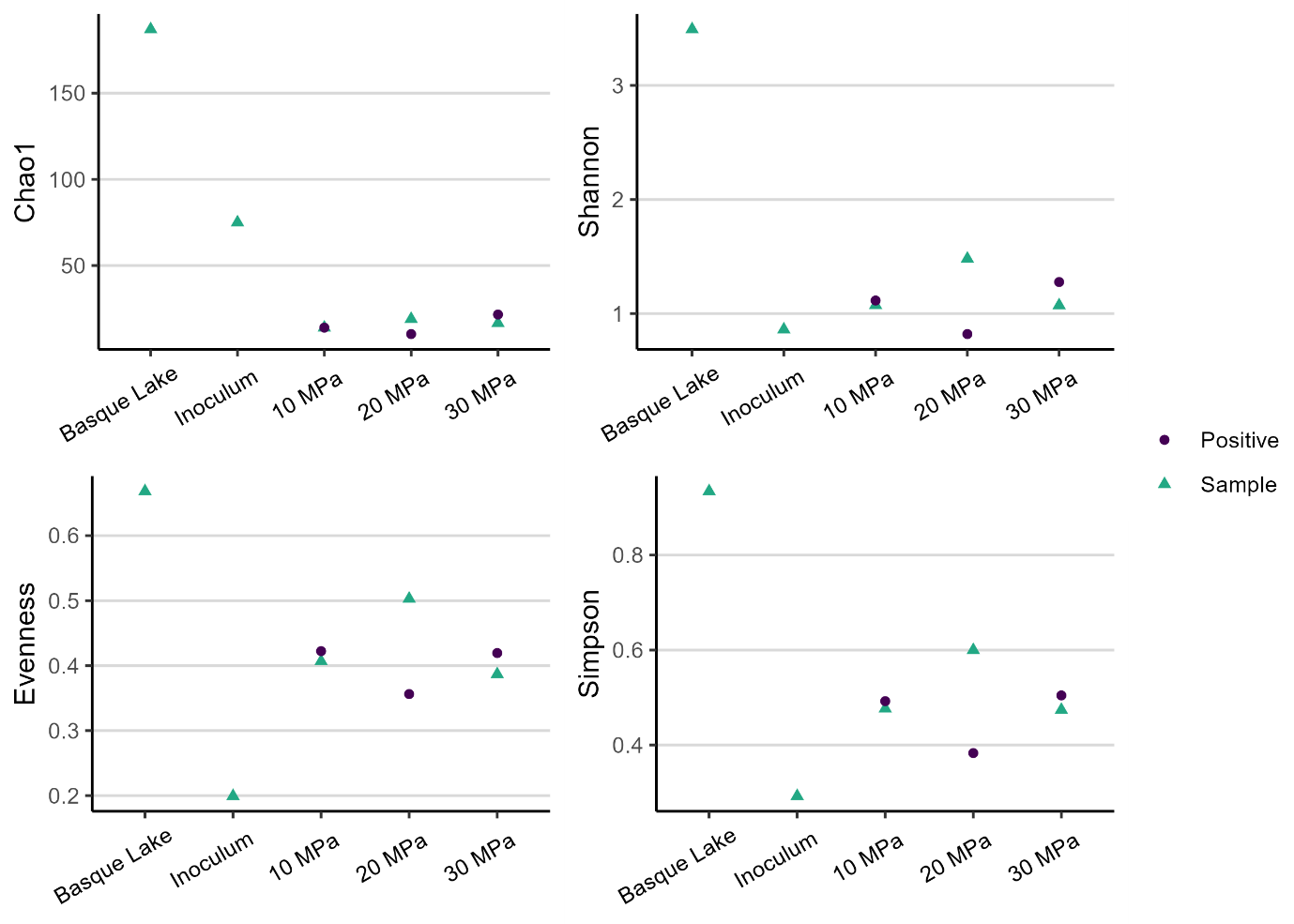
**

Supplementary Figure 3 Alpha diversity plots (Chao1 richness estimator, Shannon diversity index, Simpson and Pielou’s evenness index) showing the variation in diversity for each pressure step (positive controls in purple circles), the initial inoculum and a sample from Basque Lake.


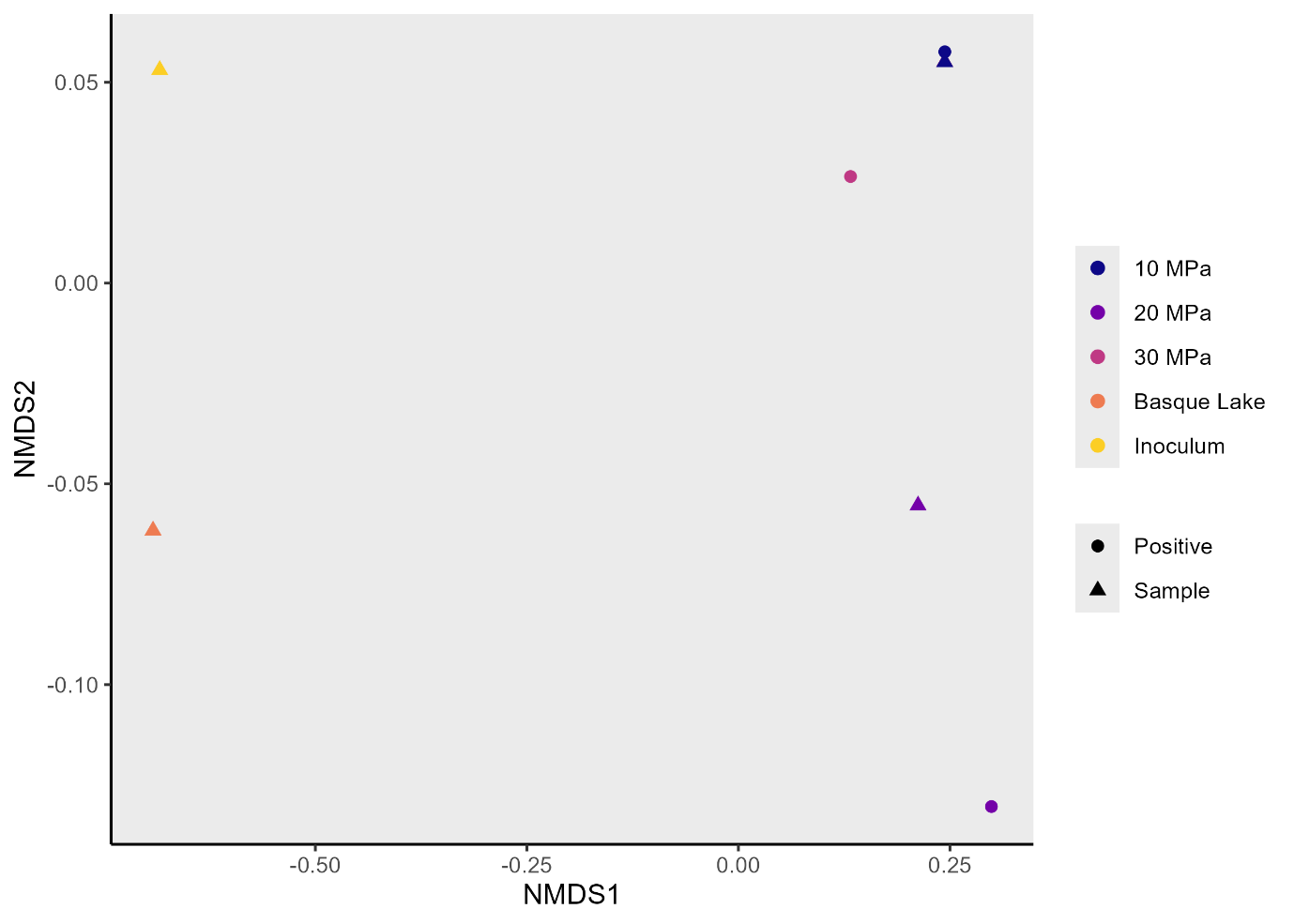


Supplementary Figure 4 Non-metric multi-dimensional scaling (NMDS) ordination plot of two axis dimensions (stress = 0.0000429) with Bray–Curtis community dissimilarities distances.

**
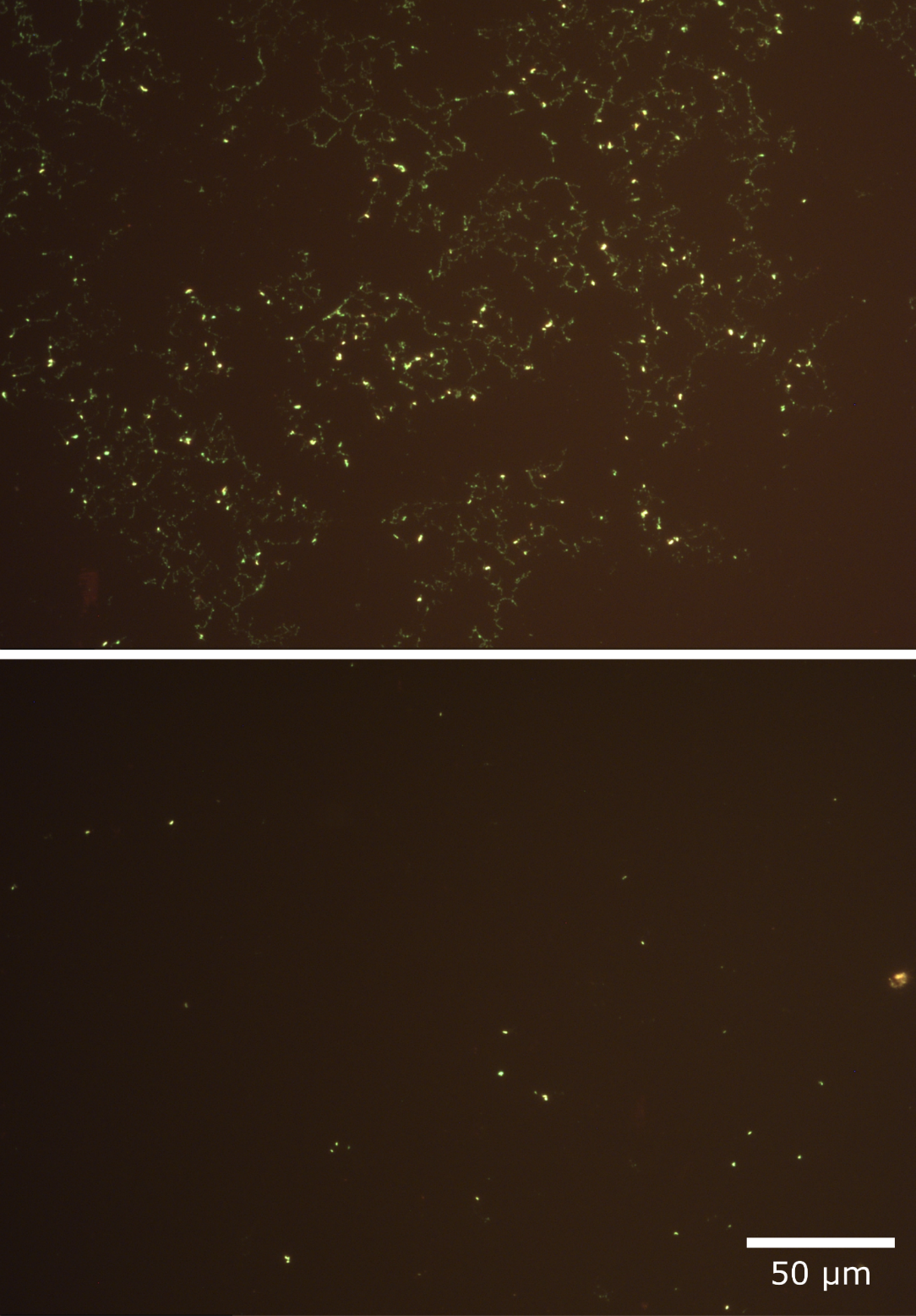
**

Supplementary Figure 5 Micrograph of acridine-orange-stained cells under a 40× magnification. Both images, (A) and (B) correspond to different regions of the same sample in the microscope slide (scale bar applies to both).
